# Some useful ideas for multistate protein design: Effect of amino acid substitutions on the multistate proteins stability and the rate of protein structure formation

**DOI:** 10.3389/fmolb.2022.983009

**Published:** 2022-08-26

**Authors:** M. A. Majorina, T. N. Melnik, A. S. Glukhov, B. S. Melnik

**Affiliations:** ^1^ Institute of Protein Research, Russian Academy of Sciences, Moscow, Russia; ^2^ Shemyakin–Ovchinnikov Institute of Bioorganic Chemistry, Russian Academy of Sciences, Moscow, Russia

**Keywords:** protein folding, amino acid substitutions, energy landscape of the protein, stability of multistste proteins, folding rate of multistste proteins, apomyoglobin, carbonic anhydrase

## Abstract

The design of new protein variants is usually confined to slightly “fixing” an already existing protein, adapting it to certain conditions or to a new substrate. This is relatively easy to do if the fragment of the protein to be affected, such as the active site of the protein, is known. But what if you need to “fix” the stability of a protein or the rate of its native or intermediate state formation? Having studied a large number of protein mutant forms, we have established the effect of various amino acid substitutions on the energy landscape of the protein. As a result, we have revealed a number of patterns to help researchers identify amino acid residues that determine the folding rate and the stability of globular proteins states and design a mutant form of a protein with desired properties.

## Introduction

The idea of being able to design a protein with desired properties has been of concern to scientists for a long time. Many tools (theoretical programs and experimental methods) that bring the possibility of *de novo* protein creation closer have already been developed. However, researchers are still limited mainly to “correction” of already known proteins.

Suppose you need to change the activity of some enzyme. The task is challenging, but at least it is clear how to undertake it. First you need to identify the active site of the protein, thus narrowing the search for important amino acids. And then - «let’s go»! You use intuition and the whole arsenal of theoretical and experimental methods to determine the mutation need to be introduced, and then verify its effect on the properties of the protein. Sometimes it is possible to apply random mutagenesis methods and select the desired (or required) mutant protein, for example, by its fluorescence. Of course, it is also desirable that the region of random amino acid substitutions be as narrow as possible.

But what if the purpose is to change the folding properties of the protein? Where are the “active sites” responsible for the folding rate, for the number of intermediate states, and for stability of these states? Unfortunately, there are no answers to these questions, since the problem of protein folding, in general, has not yet been solved. An advanced reader may object: what about the AlphaFold program (Jumper et al., 2021) Indeed, the recently developed alphaFold program is successful at predicting the spatial structure of a protein by its amino acid sequence, but still it cannot predict the stability of a protein molecule, the rate of protein folding, not to mention the stability of the intermediate states.

Is it possible to narrow that down and pre-select the amino acids responsible for the stability and rate of protein states formation?

There are books or reviews that explain the general principles of protein folding and describe the amino acids that are statistically important for the process [for example, (Finkelstein and Ptitsyn, 2016)]. There are also many complex experimental and theoretical methods for determining “important” amino acids or structural regions of a certain protein (Melnik et al., 2012; Rocklin et al., 2017; Ahmed et al., 2022).

But usually more simple information is required, such as: substitution of such-and-such amino acids will lead to such-and-such changes in the folding rate and stability of the protein. Particular recommendations are difficult to find in the literature. It is generally recommended to use some experimental methods to determine the desired mutation or a program predicting the probable effect of mutations on the protein. Each of these programs or experimental techniques has its own limitations. There are no universal recommendations for the simple reason that the problem of protein folding is still not solved, despite significant progress in recent years, both in experimental and theoretical methods.

In the search for amino acids that affect the protein folding rate, the φ-value analysis was invented (Matouschek and Fersht, 1991; Fersht and Sato, 2004; Salvatella et al., 2005). Alan R. Fersht, the author of this method, developed an experimental approach to determine the amino acids within the so-called folding nucleus. The approach involves investigation of a large number of protein mutant forms. As a result, it is possible to identify hydrophobic amino acids that affect the energy barrier separating the native and unfolded states of the protein. That is, to find the amino acids “responsible” for the protein folding rate. Several proteins have been investigated using this approach (Otzen et al., 1994; Nolting, 1999; Campos et al., 2004; Inobe and Kuwajima, 2004; Paci et al., 2004; Salvatella et al., 2005; Petrovich et al., 2006; Sato et al., 2006; Samatova et al., 2010; Zarrine-Afsar et al., 2010). In the sequence of each studied protein, amino acids that affect the folding rate (folding nucleus amino acids) were identified. But unfortunately, these results cannot be used to formulate a general rule that will allow us to predict which amino acids in a globular protein are responsible for the protein folding rate.

The φ-value analysis was developed for two-state proteins folding in one step (which required no stable intermediates for complete and successful folding). However, the idea of comparing the energy landscapes of wild-type proteins and their mutant forms to identify “important” amino acid residues is applicable to multistage proteins as well. The experimental methodology for studying one-step and multi-step folding proteins is slightly different.

**FIGURE 1:**
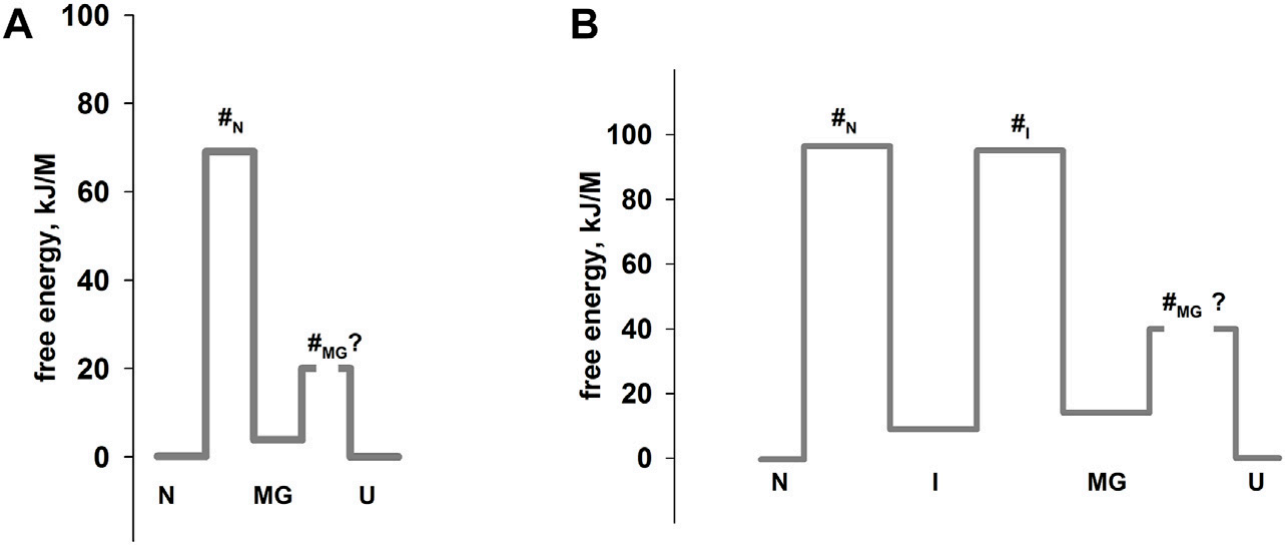
Free energy profiles for apomyoglobin **(A)** and carbonic anhydrase **(B)**. N, native state; I, intermediate state; MGi, molten globule intermediate state; #, transition state; U, unfolded state. The free energy profiles shown in the figure were calculated for conditions when native and unfolded states of proteins are equal in their stability. For apomyoglobin it is 11°C, pH 6.2, 2.7°M urea concentration (Baryshnikova et al., 2005b) [ref], for carbonic anhydrase 20°C, pH 8.0, 6.2°M (Melnik et al., 2008) [ref]. The question mark next to #_MG_ means that the rate of molten globule state formation cannot be measured experimentally.

Оur previous article (Baryshnikova et al., 2005b) described in detail an experimental approach to studying multi-state protein folding, which occurs *via* an intermediate state of a molten globule type. This method accommodates the influence of the fast folding phase (its rate constant cannot be measured) on the subsequent slow stages of protein folding. This approach has been successfully used for two proteins: sperm whale apomyoglobin (PDB:1bzp) and bovine carbonic anhydrase II (PDB:1v9e) (further in the text, just: apomyoglobin and carbonic anhydrase) (Baryshnikova et al., 2005b; Samatova et al., 2010; Melnik et al., 2014). This method provides calculation of the true (not the visible) rate constants of protein folding and unfolding, estimation of the stability of a rapidly forming intermediate states and, accordingly, depicting of the protein energy landscape. A slice of such a landscape under specific conditions is more commonly referred to as an energy profile. Figure 1 shows the free energy profiles for apomyoglobin and carbonic anhydrase.

It is worth emphasizing that the experimental approach (chevron plot analysis) makes it possible to analyze the energy landscape of the protein over the entire range of conditions (usually, at various denaturant concentrations). However, energy profiles are usually calculated at certain “points” of the landscape—for example, when the free energy of the native and unfolded states is equal (Figure 1) or when the free energy of adjacent states (native and intermediate) is equal.

Thus, our approach makes it possible to calculate free energies of almost all protein states. Using this method, we investigated the effect of various amino acids substitutions on apomyoglobin and carbonic anhydrase energy landscapes and identified groups of amino acids that, being substituted, affect different states of the protein.

## Results and discussions

### Single amino acid substitutions in any region of the protein do not affect the stability of molten globule intermediate state of the protein

With ready-made research results, we can say the classic phrase: “it was obvious.” Indeed, the first intermediate state of a protein in the process of its folding, a molten globule, is characterized by the formation of secondary structure elements, but lacking the specific for native protein tight packing of amino acid residues (Finkelstein and Ptitsyn, 2016). Therefore, it seems logical that the substitution of any amino acid residue should not affect the stability of a mobile, non-rigid intermediate state such as a molten globule. However, without experimental verification, doubts may be expressed. For example, it can be assumed that the stability of a molten globule depends on non-specific contacts of amino acids, as a certain hydrophobic core is formed at the first stage of protein folding and its structure is maintained due to the interaction of amino acid residues, even if they non-specific.

We have investigated about 20 mutant forms of apomyoglobin, and 10 mutant forms of carbonic anhydrase. Apomyoglobin and carbonic anhydrase were chosen as model proteins, since not only their native state, but also all their intermediate states were characterized (Semisotnov et al., 1991; Barrick and Baldwin, 1993; Jennings and Wright, 1993; Uverskii et al., 1993; Eliezer and Wright, 1996; Bhakuni, 1998; Garcia et al., 2000; Nishimura et al., 2003; Melnik et al., 2008; Nemtseva et al., 2017). In particular, it was shown that the molten globule state of apomyoglobin is characterized by a native-like secondary structure.

**FIGURE 2:**
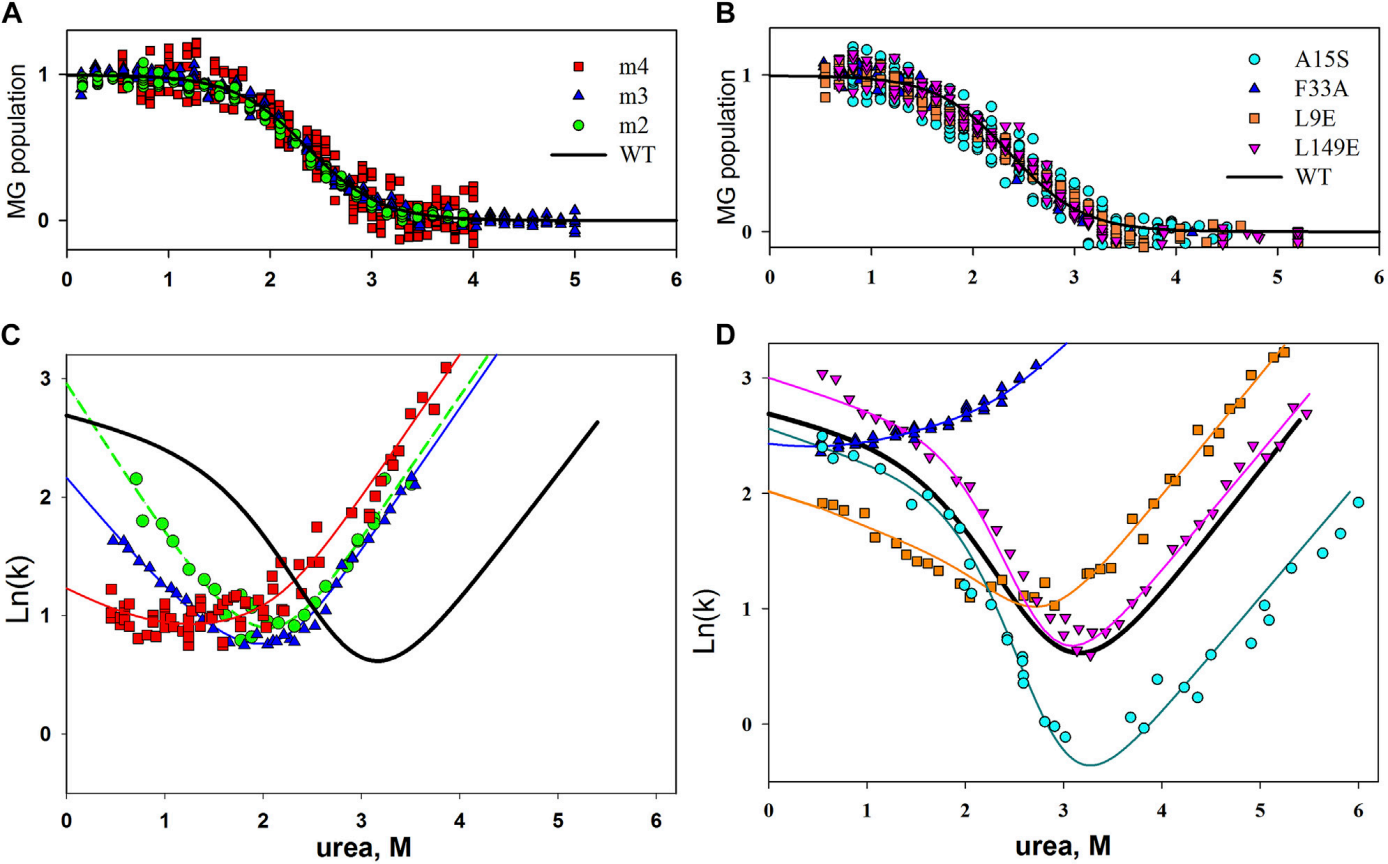
**(A**,**C)** Plot of molten globule state population versus urea concentration for wild-type and mutant apomyoglobin. **(B**,**D)** Chevron plots of wild-type apomyoglobin and its mutant form. Experimentally obtained data for mutant forms of the protein are shown by symbols; the approximation of these data with a use of parameters assuming that the substitution did not change the stability of the molten globule state is shown by lines. m2, m3 and m4, simultaneous substitution of two, three and four amino acids; m2, A15S, A19S; m3, A15S, A19S, V21T; m4, L9D, A15S, A19S, V21T.

According to the results of our studies, none of the single amino acid substitutions in the protein core and on its surface affected the intermediate state of the molten globule type. Figure 2 shows population graphs of apomyoglobin molten globule state, measured for mutant forms of this protein. It can be seen that all the curves coincide within the experimental error. In addition, multiple substitutions of 2, 3, 4 and 6 amino acid residues on the surface of the apomyoglobin molecule have been investigated. We assumed that if single substitutions of amino acid residues from the protein surface have minor effect on the molten globule state, then this effect may be enhanced with a rising number of substituted residues. The results are shown in Figure 2B. It can be seen that substitution of several amino acid residues also did not affect the stability of the molten globule state.

The same conclusion can be drawn by analyzing chevron plots, since their shape depends on the stability of the protein intermediate state, which is formed during the dead time of the stopped-flow instrument. The analysis of chevron plots is described in detail in (Baryshnikova et al., 2005b; Melnik et al., 2008), but in a simplistic way, it is necessary to make a chevron plot approximation with parameters assuming that the stability of the molten globule intermediate remained the same as that of the wild-type protein. If the chevron plot is well approximated, this means that the mutation did not affect the intermediate state. If the chevron plot is poorly approximated, then the stability of the intermediate state has changed. The result is shown in Figure 2C. It can be seen that the experimentally obtained chevron plots differ from each other, and yet they are well approximated by parameters that suggests that the stability of the intermediate state of apomyoglobin has not changed.

Thus, we may conclude that it is practically impossible to affect an molten globule intermediate state by replacing individual residues.

The result seems puzzling since many proteins with varying stability of intermediate states are known, and therefore there must be ways to influence this stability. We suggested that the stability of an intermediate state such as a molten globule may depend on the flexibility of the protein chain. The flexibility of protein chains is provided by: *1*) loops connecting the elements of the secondary structure; *2*) prolines and glycines in the amino acid sequence; *3*) cysteine bridges—covalent bonds between distant amino acid residues (cysteines).

### The molten globule intermediate state of a protein can be stabilized by introducing cysteine bridges into the protein structure

To test our hypothesis, we designed a cysteine bridge in apomyoglobin.

**FIGURE 3:**
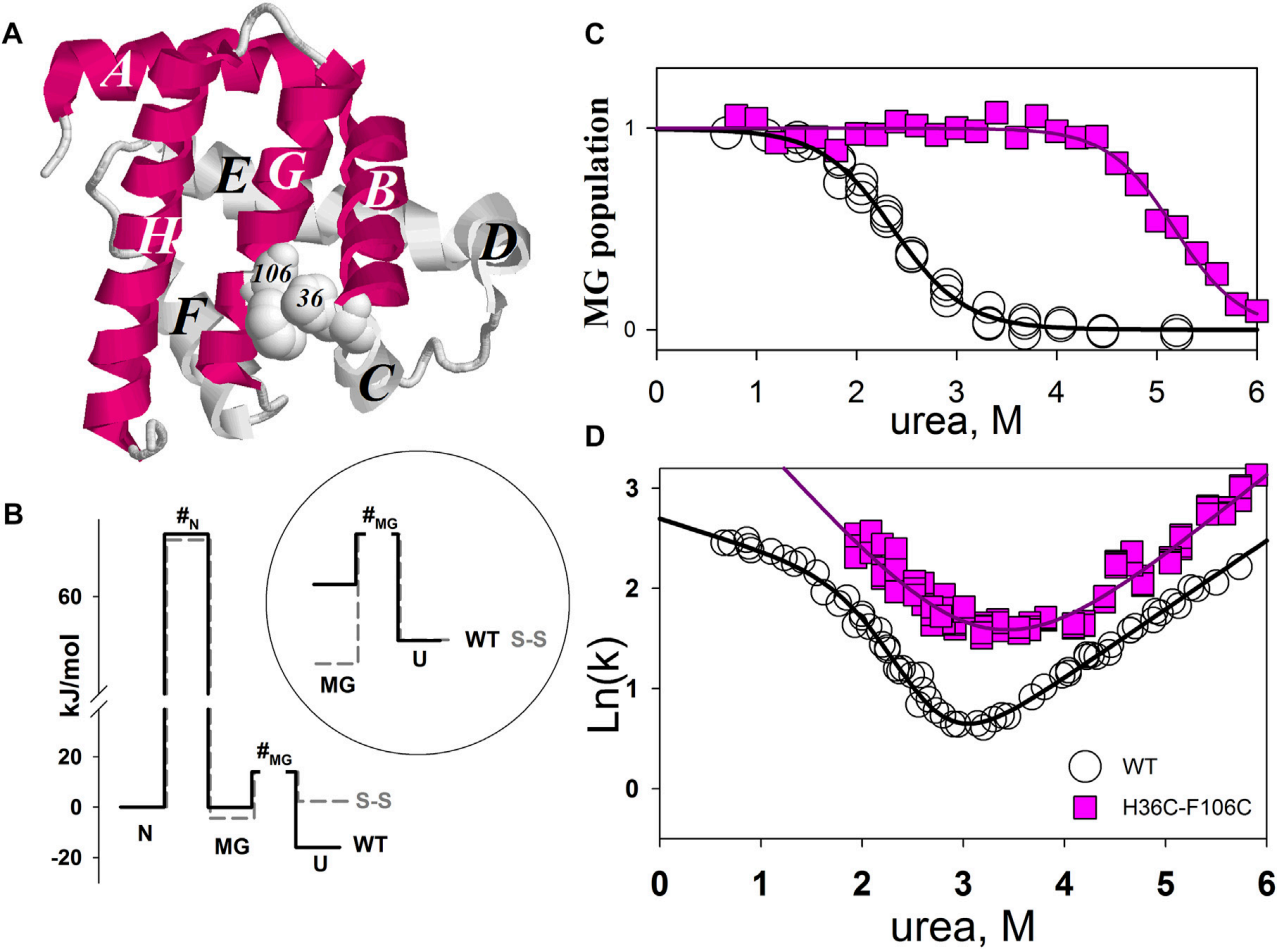
**(A)** 3D structure of myoglobin. Letters from A to H identifies α-helices. α-helices folded first (Jennings and Wright, 1993) are shown in red. Amino acids H36 and F106 that have been substituted by cysteines are shown in volume. **(B)** Free energy profiles of wild-type (WT) and mutant (S-S) apomyoglobin calculated from molten globule state population plots **(C)** and chevron plots **(D)**. N, native state; I, intermediate state; MG, molten globule intermediate state; #, transition state; U, unfolded state. Free energy profiles calculated for proteins at 4.3 M urea, 11°C, pH 6.2 (Baryshnikova et al., 2005b). There is always some arbitrariness in comparing the energy profiles of different proteins with each other, since it is impossible to calculate the absolute values of free energies, only their relative values are calculated. The cysteine bridge changes the entropy of the protein unfolded state and, accordingly, increases the free energy of the unfolded state, so we assumed that a comparison of the free energy profiles aligned with the native state would provide a clearer understanding of changes in the energy landscape of mutant apomyoglobin. Additionally, the circle shows a part of the free energy profile (transition between MG and U states) aligned with the unfolded state.

Figure 3A shows the structure of myoglobin (without heme) and the positions of amino acid residues substituted for cysteines. The population of the intermediate molten globule state and the folding/unfolding rate at different concentrations of urea were measured for the mutant form of the protein. As a result, we were able to calculate the free energies of different protein states and obtain a free energy profile. Figure 3B shows the free energy profile for wild-type apomyoglobin and its mutant form.

Intermediate state population plots and chevron plots are shown in Figures 3C,D. It can be seen that the introduction of a cysteine bridge into the protein structure shifted the population curve of the apomyoglobin intermediate state by almost 3 mol of urea (Figure 3C). This is an indication of significant increase in the stability of the molten globule state (MG) relative to the unfolded protein state (U). The inset in Figure 3B shows the MG and U energy levels aligned with the unfolded state. The pronounced stabilization of the MG state is partly explained by the destabilization of the U state (Figure 3B), but the MG state also stabilized relative to the native state (N) (Figure 3B). The cysteine bridge “fastens” the polypeptide chain and reduces the entropy of the unfolded state. This effect was predicted in theoretical works and shown experimentally for two-state proteins (Matsumura et al., 1989; Vogl et al., 1995; Nikitina et al., 2008). It is worth noting that if a cysteine bridge is introduced into a multistate protein, it is more correct to compare the energy profiles aligned with the free energy of the native state as in Figure 3B, but in some cases, transitions between adjacent protein states must be considered separately.

Although the ss-bridge stabilized the MG states, the minimum of the chevron plot remained virtually unchanged. The minimum of the chevron plot corresponds to the urea concentration at which the process of protein folding turns into unfolding. That is, the position of the minimum related the stability of the native state (N). Why, then, did the stabilization of MG not lead to the stabilization of the native state? The matter is that the cysteine bridge significantly changed the energy landscape of the protein. For the wild-type protein, the main states are N and U, since they are the only populated under equilibrium conditions (the MG state was detected only in kinetic experiments). In the mutant protein, the three states N, MG, and U were populated.

**FIGURE 4:**
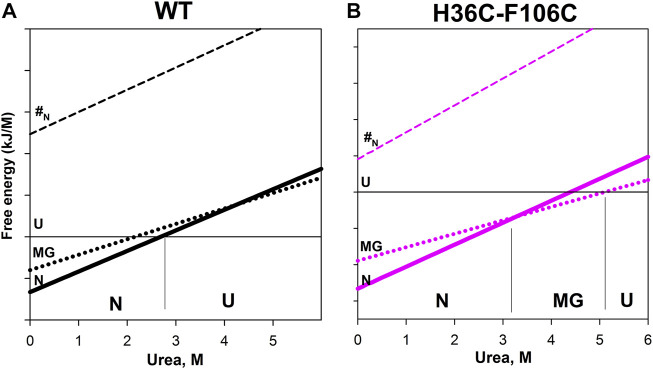
Free energy dependence of native state (N), molten globule intermediate state (MG), unfolded state (U), and transition state (#N) for wild-type apomyoglobin **(A)** and its mutant form with cysteine bridg **(B)**. The cut of such a dependence under certain conditions is the free energy profile. For example, for wild-type apomyoglobin at 2.7 M urea, the free energy of the N state is equal to the free energy of the U state—the profile for such conditions is shown in Figure 1. Near the lower axis, the letters N, MG and U indicate the most stable state at given urea concentrations. The division on the vertical axis corresponds to 20 kJ/M.

Figure 4 shows the dependencies of free energy of all states (N, MG, U, and #-transition state) of wild-type apomyoglobin and its mutant form with an integrated cysteine bridge on urea concentration.

The plot enables us to “see” all the free energy profiles for all conditions at once. For example, the free energy profile shown in Figure 1A corresponds to the points in Figure 4A at a concentration of 2.7 M (free energy equality for N and U states), and the profiles shown in Figure 3 correspond to the points in Figures 4A,B at a concentration of 4.3 M (free energies of N and MG states of the wild-type protein are equal).

It can be seen that the introduction of the cysteine bridge affected the U and MG states (for MG the line went down, and for U it went up). At low urea concentrations, the most stable state of the wild-type protein is the native (Figure 4A), and after increasing the urea concentration to 3 M –unfolded state (Figure 4A). The energy landscape of the mutant protein looks different (Figure 4B). In the range from 0 to 3.3 M urea, the native state is the most stable, then (at a urea concentration of 3.3–5.1 M) the intermediate state MG, and only after increasing the concentration to 5.1 M the unfolded state U becomes the most stable. Hence, the stability of the wild-type protein native state under equilibrium conditions was determined by the transition between the N and U states, and for the mutant protein, by the transition between the N and MG states.

Our result indicates that the introduction of a cysteine bridge into the structure of multistate proteins does not always stabilize the protein or may even lead to its destabilization. Under the stabilization/destabilization of the protein is usually meant the stabilization/destabilization of the native state, relative to non-native states.

Analysis and generalization of the results obtained allow us to conclude that cysteine bridges stabilize (reduce free energy) mobile, weakly structured intermediate states of the protein (a molten globule type, for example) and destabilize the unfolded state of the protein.

### Substitutions of hydrophobic amino acid residues with a large number of residue-residue contacts do not change the rate of protein folding, but may affect the rate of its unfolding

We noticed this pattern in the study of carbonic anhydrase mutant forms. It is a protein with several intermediate states, both weakly structured (molten globule) and well structured native-like. All protein states have been previously characterized (Semisotnov et al., 1991; Uverskii et al., 1993; Kumar et al., 1998).

**FIGURE 5:**
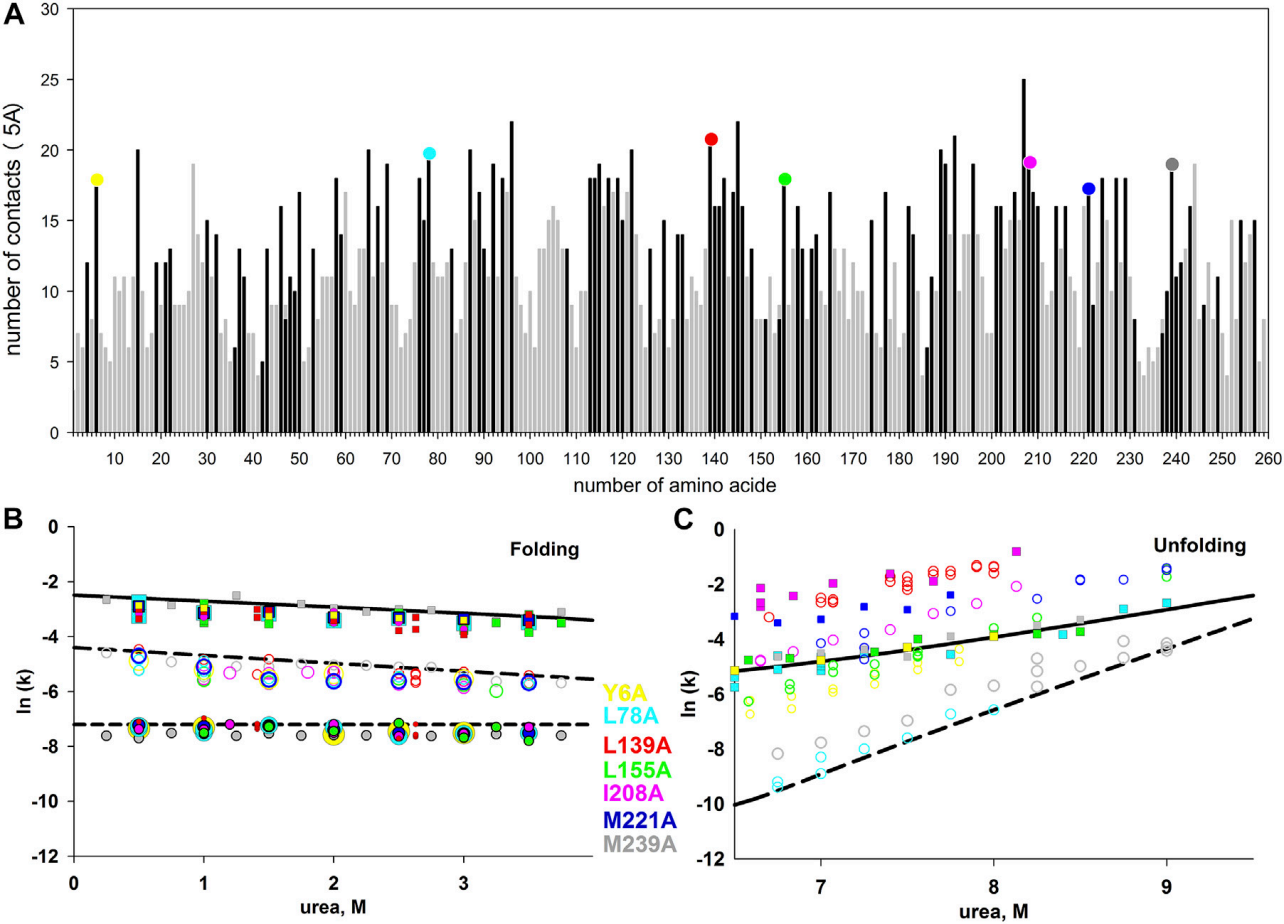
**(A)** - the number of contacts of each amino acid residue of carbonic anhydrase. Residue-residue contacts were calculated at a distance of up to 5 Å. Dark bars correspond to hydrophobic amino acids, light bars correspond to hydrophilic ones. Colored circles at the top of the columns highlight the seven studied amino acid substitutions. Figures **(B**,**C)** show the dependences of the rate constants of the folding **(B)** and unfolding **(C)** of the carbonic anhydrase mutant forms. The rate constants for the wild-type protein are shown as lines, for the mutant forms, as symbols.

We chose for substitution seven hydrophobic amino acid residues of carbonic anhydrase with the largest number of contacts. We assumed that if the contacts between amino acid residues are “responsible” for the formation of some states, then the substitutions of amino acid residues with the largest number of residue-residue interactions should maximally affect the rate or stability of protein states. Residue-residue contacts of bovine carbonic anhydrase II (PDB: 1v9e) amino acid residues are shown in Figure 5.

However, the results of kinetic measurements surprised us. None of the substitutions significantly affected the rate of protein folding. In the process of the native state formation, carbonic anhydrase passes through at least three intermediate states. The rates of three protein folding and two unfolding stages can be measured by tryptophan fluorescence.

The dependences of folding and unfolding rate constants for the seven investigated mutant forms of proteins versus urea concentration are shown in 5B and 5C. It was found that all the studied substitutions destabilized carbonic anhydrase (according to the equilibrium unfolding of the proteins (Melnik et al., 2016; Nemtseva et al., 2017)), but most surprisingly, none of these substitutions affected any of the three stages of carbonic anhydrase folding.

As illustrated in 5B and 5C the studied amino acid substitutions differ in their effect on the unfolding rates, while the folding rates are in good agreement with those for the wild-type protein (considering the experimental error). Therefore, the selected amino acids are important for the stability of the native and intermediate states of the protein, but do not affect the energy barriers that determine the rates of protein states formation.

**FIGURE 6:**
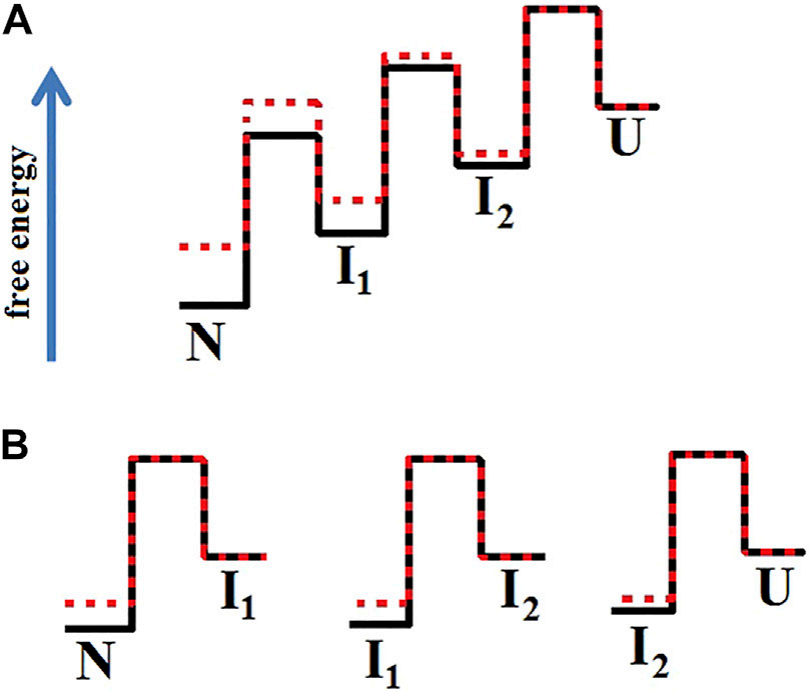
Energy profiles of two proteins when the mutation affected only the native and intermediate states stability. In Figure **(A)**, the profile of the wild-type protein (red line) aligns with the energy profile of the mutant protein (black line) in the unfolded state. Figure **(B)** separately matches each of the transitions between two adjacent states. Figure **(B)** shows that the height of the folding barriers (U→I_2_ →I_1_→N) for the wild-type protein and its mutant form did not change. This is how the energy landscape for the proteins shown in Figure 5 should look like.

Figure 6 shows an example of how mutations affect the protein energy profile. The free energy profiles of one-step folding proteins are simple (not shown in the figure), so the effect on stability is clearly seen. For multistate protein, the analysis of substitutions effect is more complicated, therefore it is more correct to compare and analyze each stage of folding/unfolding separately.

The result obtained in the study of carbonic anhydrase led us to assume that such an influence may be a general pattern for all proteins. Therefore, we decided to test the hypothesis that amino acid residues with a large number of contacts affect mainly the stability of the protein. This statement can be reformulated in terms of Fersht’s φ -analysis: the greater the number of contacts of an amino acid residue, the smaller the value of the φ parameter. Simplistically, the φ can be considered as a parameter that determines the effect of mutation on the folding rate (1—pronounced effect, 0—no effect).

**FIGURE 7:**
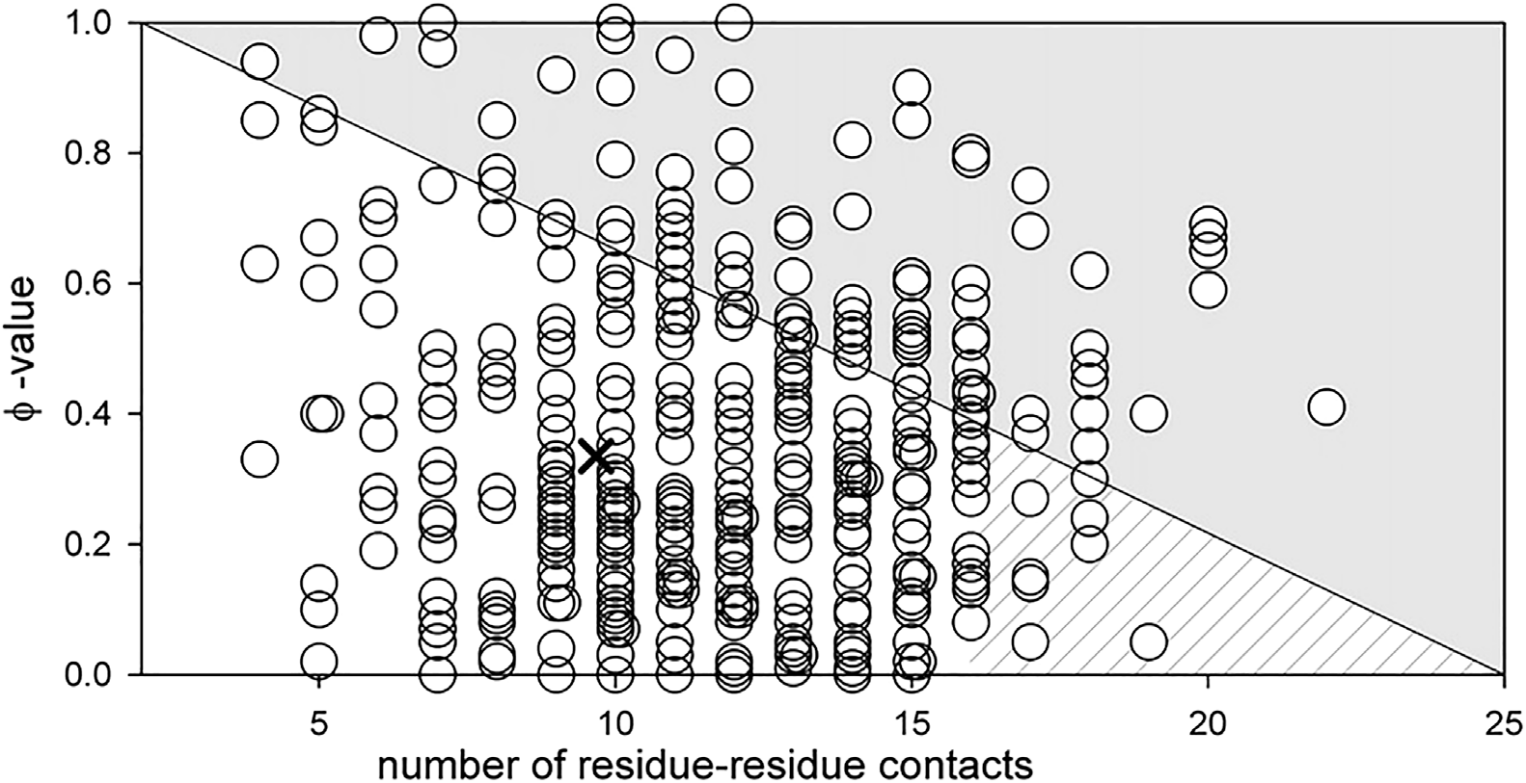
Dependence of parameter φ on the number of residue-residue contacts for an amino acid residue (*see* (Melnik et al., 2016)). The cross shows the middle of the lower white triangle, which according to the above assumption should correspond to the average value of parameter φ for all amino acid residues. Its position at φ = 0.3 is well compatible with the data reported in (Sanchez and Kiefhaber, 2003) where the average value of parameter φ of amino acids from different proteins was calculated (the average value of φ varies from 0.2 to 0.37 for different proteins). The hatched part of the white triangle shows the range of parameter φ values for amino acids with the number of contacts exceeding 15.

The graph in Figure 7 was ploted as follows. We evaluated residue-residue contacts of proteins described in the literature (Melnik et al., 2014). Each amino acid residue contacts a minimum of two adjacent amino acids in the chain and a maximum of 25 (tryptophans in the hydrophobic core). The φ parameter according to Fersht’s theory can vary from 0 to 1. We do not know what should ideally be the dependence of the φ parameter on the number of contacts (linear or other type). But the concentration of the circles cloud in Figure 7 mainly in the lower white triangle, means the following: for amino acid residues with a large number of contacts, for example, more than 15, the φ parameter will be on average about 0.2. For amino acid residues with a small number of contacts, the φ parameter can have any value from 0 to 1.

This means that large amino acid residues within the hydrophobic core are “responsible” only for the stability of native and tight packed (native-like) intermediate states, but not for the rate of their formation. This statement is confirmed by the works of Finkelstein, who showed that the folding nucleus of the protein (amino acids that determine the rate of protein folding) should be located on the edge of the protein, but not in its middle (Finkelstein and Badretdinov, 1997).

### Substitutions of amino acid residues on the protein surface may affect the rate of protein folding

**FIGURE 8:**
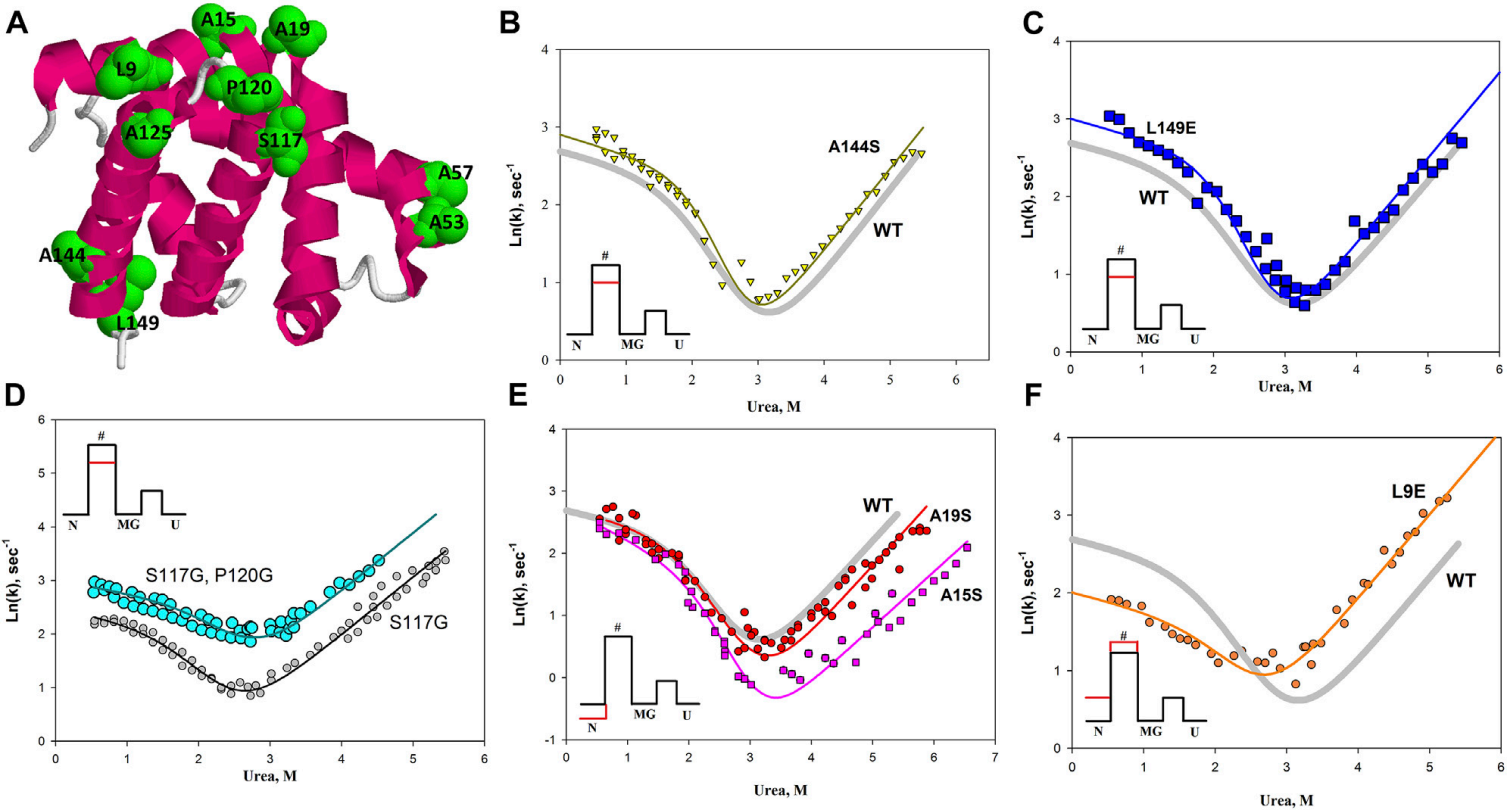
**(A)** Spatial structure of apomyoglobin. Amino acids on the surface of apomyoglobin used in the study are highlighted in green and in volume. **(B**–**F)** The chevron plot for the wild-type protein is shown as a solid gray line without symbols in the Figures. The chevron plots of the mutant proteins are shown as symbols; the colored solid lines show approximations of the experimental data. The insets show the free energy profiles, where the red line shows the effect of mutations on the free energy profile of the protein.

Based on the studies described above and the work of Finkelstein (for example Finkelstein and Badretdinov, 1997), we proposed the hypothesis that amino acid residues important for the formation of the energy landscape of the protein are located on its surface. We investigated several hydrophobic amino acid residues located on the surface of apomyoglobin. In the opening stage of research, we chose to replace hydrophobic amino acids that were maximally exposed to the solvent. Figure 8A illustrates a spatial model of apomyoglobin with the studied amino acids. It can be seen that all of them are on the surface of the protein and are maximally accessible to the solvent. Figures 8B–F shows chevron plots of the most interesting mutant proteins. Approximately half of the amino acid substitutions we studied on the surface of apomyoglobin (some not discussed in this work) gave an “interesting” result—either protein stabilization or an effect on the folding rate. It is noteworthy that among the 12 studied amino acids of the hydrophobic core of the protein, only one “interesting” substitution was found (φ = 0.7); other substitutions led to strong destabilization of native state and did not affect the folding rate.

Table 1 shows the values of the φ parameter and changes in the logarithm of the folding and unfolding rate constants for the studied proteins (shift of the corresponding branch of the chevron plot).

**TABLE 1:** Result of chevron plot analysis of amino acid substitutions on the surface of apomyoglobin.

Substitution	Δln (k_IN_)	Δln (k_NI_)	φ ± 0.2
L9E	0.7 ± 0.1	−0.9 ± 0.2	0.5
A15S	0.1 ± 0.1	1.3 ± 0.2	−0.1
A19S	0 ± 0.2	0.4 ± 0.2	−0.1
A53S	−0.05 ± 0.04	−0.12 ± 0.04	—
A57S	0.1 ± 0.04	−0.5 ± 0.1	0.2
A125S	0.05 ± 0.04	0 ± 0.2	—
A144S	−0.2 ± 0.1	−0.3 ± 0.1	2.0
L149E	−0.3 ± 0.1	−0.2 ± 0.1	1.4

Δln (k_IN_), shift of the chevron plot folding branch; Δln (k_IN_), shift of the chevron plot unfolding branch, φ is the Fersht parameter. The values of the φ for the mutant forms of apomyoglobin with the A53S and A125S substitutions were not calculated, since the chevron plots of these proteins differ slightly from the chevron plot of the wild-type protein.

The most interesting result for us was the effect of amino acid substitutions exclusively on the energy barrier of the protein (for apomyoglobin, the barrier separating the native state and the state of the molten globule type). The A144S and L149E mutations raised both the fold and unfold branches of the chevron as shown in Figures 8B,C. Therefore, the mutation lowers the energy barrier in both folding and unfolding and does not affect the N and MG states, but only the energy barrier between them. Double amino acid substitution in the protein led to a similar result (Figure 8D). In the study of amino acid substitutions that could lead to an increase in the flexibility of the protein backbone (S177G and P120G substitutions), it was found that the P120G substitution in a protein with the already introduced S177G substitution significantly increases the rate of both protein folding and unfolding (Figure 8D). That is, the additional P120G substitution affects the energy barrier without affecting the N and MG states.

The insets of Figures 8B–D show the changes in the energy profile of the protein with this effect of amino acid substitutions on the chevron plot.

In addition, we found that the A15S and A19S substitutions stabilize the native state of apomyoglobin, and the L9E substitution, despite the destabilization of the native state, also slows down the rate of protein folding, i.e. increases the energy barrier between native and molten globule states.

What conclusions can be drawn from the results described above and from previous studies of hydrophobic amino acids substitutions in the apomyoglobin hydrophobic core?

It can be concluded that some protein amino acids are responsible for the free energy of tight-packed states (native and intermediate), and completely different ones - for the free energy of transition state (a virtual state located at the top of the energy barrier).

The stability of the tight-packed native state of a protein depends mainly on the hydrophobic amino acid residues in the hydrophobic core of the protein. Any substitution of such amino acids leads to a strong destabilization of the protein, but does not significantly affect the rate of folding (*see* the previous chapter and (Dill et al., 1993; Gerstman and Chapagain, 2005; Finkelstein and Ptitsyn, 2016)).

The protein folding rate (in other terminology: the height of the energy barrier, the stability of the transition state) depends on the amino acids on the surface of the protein. This statement is obviously devoid of specificity. Can we clarify which amino acids affect the rate of protein folding? Unfortunately, not. Both hydrophilic and hydrophobic amino acids on the protein surface were substituted during the study, but we did not reveal a clear pattern. But these studies helped to verify another assumption.

**FIGURE 9:**
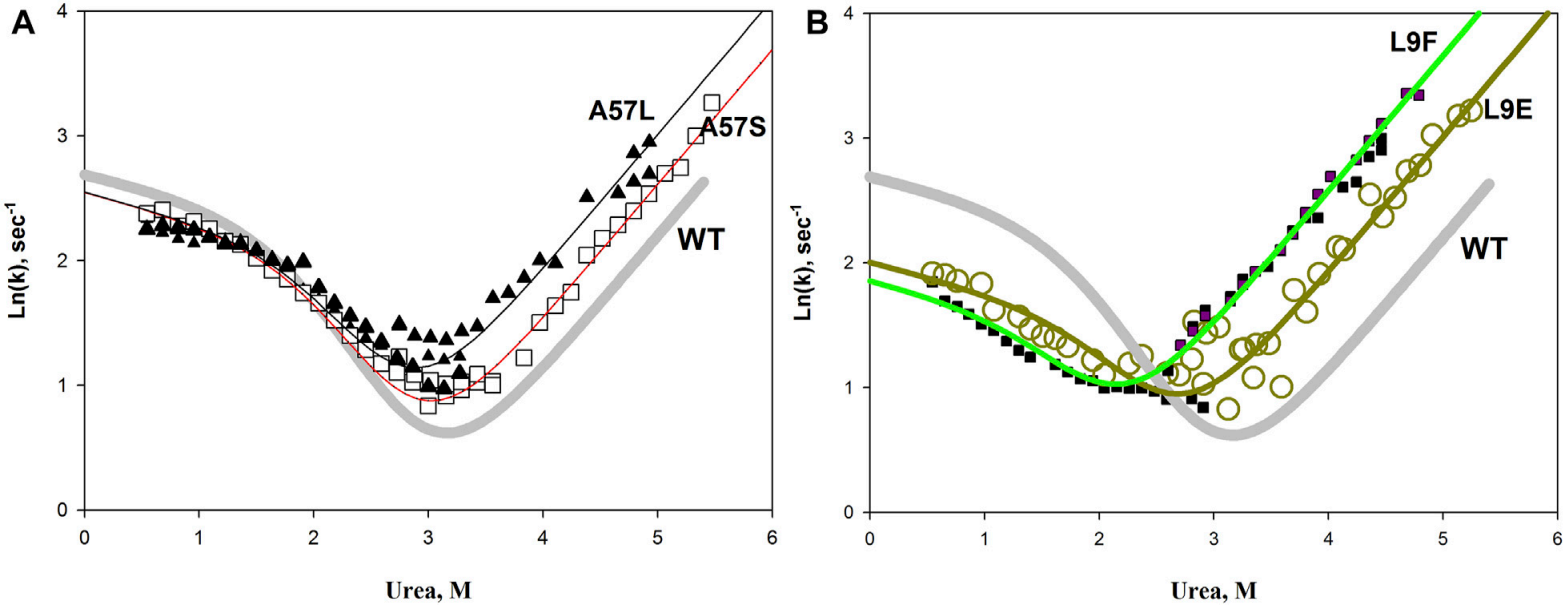
Chevron plots of wild-type apomyoglobin (thick gray line) and proteins with A57L, A57S substitutions **(A)**, and L9F L9E substitutions **(B)**. Symbols show experimental data, lines show approximations according to the three-state model.

We hypothesized that an increase in the hydrophilic surface should stabilize the protein. In general, this assumption turned out to be correct, but, unfortunately, in a particular protein, the pattern can be violated. For example, Figure 9 shows the results of replacing the same amino acid on the protein surface with hydrophobic and hydrophilic amino acids. It can be seen that a simple substitution, for example, L9E or A57S, does not provide protein stabilization. Both mutant proteins are destabilized relative to the wild type protein. However, if we compare mutant proteins with each other, for example, A57L and A57S, we can see that the protein with the A57S substitution is more stable than A57L (the chevron unfolding branch for A57S is lower than for A57L). The same conclusion can be drawn if we compare the L9F and L9E substitutions. The influence of the general hydrophilicity or hydrophobicity of surface amino acids on the stability of a protein is certainly noticeable, but it is insignificant compared to the disturbances in protein structure that can be caused by substitution of a particular amino acid in a protein. Thereby, it is impossible to change only the hydrophobicity without affecting the interactions, volume, flexibility of the chain.

### Experimentally established effects of amino acid substitutions on the protein energy landscape

Systematic studies of apomyoglobin and carbonic anhydrase allowed us to formulate several patterns that, in our opinion, can be useful in the study of soluble globular proteins or in the design of such proteins. The table below summarizes these conclusions (in a simplified way), and also schematically shows the type of mutation and its effect on the energy profile of the protein.

The conclusions given in Table 2 do not solve all the problems of designing proteins with desired properties, but in most cases they can narrow the search for amino acid residues associated with the target property of the protein.

**TABLE 2:** Effects of amino acid substitutions on protein free energy profile.

	Schematic representation of a protein and substitutions	Experimentally established effects of substitutions on protein	Effect of substitutions on protein free energy profile, shown schematically
1	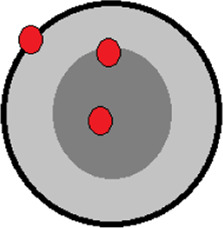	Single amino acid substitutions in any region of the protein do not affect the stability of the molten globule Melnik et al., (2014).	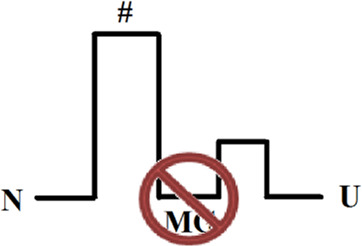
2	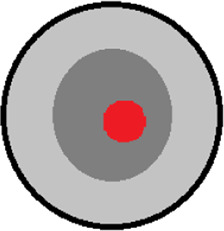	Substitutions of amino acids with a large number of contacts destabilize the native or native-like intermediate states of the protein, but do not affect its folding rate Melnik et al., (2016).	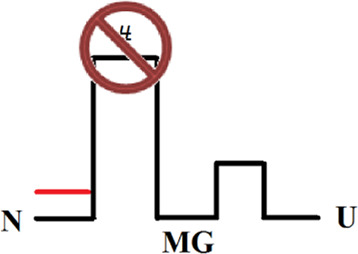
3	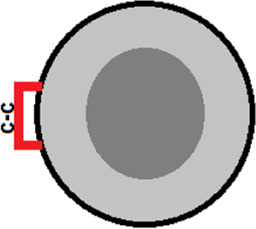	The S-S bridge on the surface of the protein stabilizes the molten globule intermediate state and destabilizes the unfolded state of the protein Melnik et al., (2014).	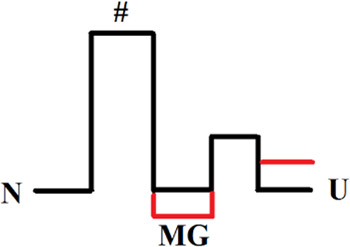
4	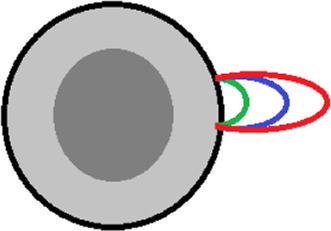	Elongation/shortening of loops on the protein surface destabilizes the state of the molten globule type relative to the unfolded state of the protein Majorina et al., (2020).	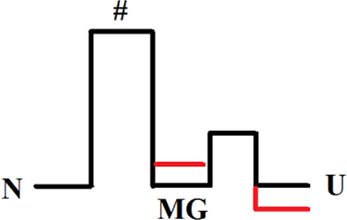
5	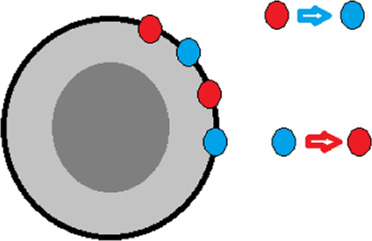	Substitution of hydrophobic amino acids on the surface of a protein with hydrophilic ones may stabilize the protein Majorina et al., (2018).	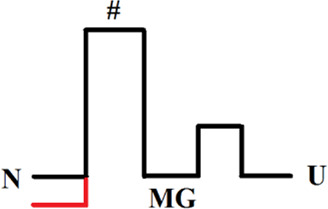
6	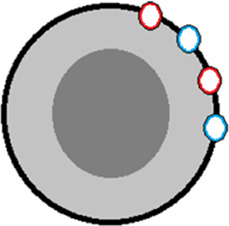	On the protein surface, amino acids that can affect the energy barrier without affecting the stability of the native and intermediate states may be found Majorina et al., (2018).	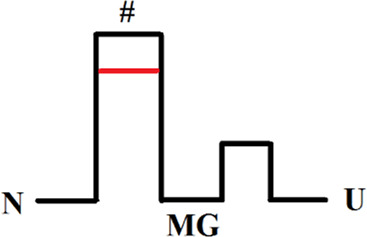

The protein is shown schematically as a gray circle, the hydrophobic core is highlighted in dark. Substitutions are shown as colored circles or lines.

For example, it is useless to introduce cysteine bridges in an attempt to create a more stable variant of the protein with intermediate state of a molten globule type (MG), since the native state (N) may be destabilized relative to the MG state (Table 2 row 3). For such a protein, most likely, an integrated approach is required—destabilization of the unfolded state (U) by the introduction of a cysteine bridge, destabilization of the intermediate state (MG), for example, by mutations in loops, and stabilization of the native state (N), for example, by salt bridges.

An interesting suggestion can be made for intrinsically disordered proteins. Since the native state of these proteins is flexible and weakly structured (similar to a molten globule state) single amino acid substitutions apparently will not affect such a protein, but the introduction of prolines, glycines or cysteine bridges in their structure may radically change their stability or function.

Our research may also contribute to the understanding of protein folding during macromolecular crowding in the cell. It can be assumed that if protein-protein interactions do not lead to aggregation, then a high protein concentration can affect the folding process approximately in the same way as the cysteine bridge introduced by us during the study—stabilize the MG intermediate state and destabilize U state.

If the task is to change the protein folding rate, you can refer to paragraph 2 of Table 2: select the amino acid residues on the protein surface without changing the hydrophobic amino acids in the hydrophobic core of the protein. Such advice may seem too vague, but for a small protein (100–200 amino acid residues) there are not so many amino acid residues on the surface. The number of selected amino acids can be significantly reduced by excluding those that are important for the functioning of the protein or conservative for this group of proteins.

One more example. The task is to find amino acid residues that affect the apomyoglobin folding rate. There are 153 amino acid residues in apomyoglobin, 37 on its surface. Of the last group, only nine hydrophobic residues have a solvent-accessible surface area of more than 55 Å (the average value of the available amino acid surface area in apomyoglobin). One of these amino acids is in the F-helix, which is unfolded in the Apo form of this protein, so we exclude it from the study. Only eight amino acid residues remain. This is how we chose the eight amino acids shown in Table 1. After analyzing the results of these eight amino acids substitution, we selected two of them (A144S and L149E), which increase the rate of apomyoglobin folding. If the task was to stabilize the protein, then we would also solve it—the A15S and A19S substitutions stabilized the native state of apomyoglobin.

Thus, despite the fact that the rules described in Table 2 may seem too vague, they significantly narrow the search for amino acids that are important in creating a protein with altered properties, such as a given stability of the native or intermediate states or the rate of the native state formation.

A few words about the correlation between protein stability, its folding rate, and functional activity. This question is always of interest to biotechnologists. Often an attempt to influence one property of a protein negatively affects another. However, the results of our research are encouraging. As it turned out, it is possible to find amino acids that affect selectively the stability of the protein native state (for example A15S) or the protein folding rate (for example A144S). This means that the various properties of a protein are not as tightly interrelated as it might seem. And this, in turn, means that it is possible to find such a set of amino acid substitutions that will allow us to create a protein with the desired properties.

## Materials and methods

### Carbonic anhydrase

The bovine carbonic anhydrase II (BCAB) coding sequence was reverse-transcribed from total RNA of bone marrow cells. cDNA was synthesized using gene specific oligonucleotide primer 3′-CA2 (5′-ttt​gtc​gac​GGC​CAG​TTC​ACC​AAG​TGG​ACT​TGT​G-3′ (SalI restriction site underlined) and M-MuLV reverse transcriptase (Fermentas, Lithuania). The products of first strand synthesis were amplified using the polymerase chain reaction and a gene specific oligonucleotide primer 5′-CA2 (5′-tac​ttt​tca​tAT​GTC​CCA​TCA​CTG​GGG​ATA​C-3′ (NdeI restriction site underlined). The amplified BCAB II gene was double digested with SalI and NdeI and inserted into a pET-11c_joe vector between NdeI and SalI restriction sites. The resulting plasmid was designated as pBCAB. Plasmids with the mutant BCAB genes were constructed by a standard PCR technique, using appropriate primers and a pET-28a vector as a template and using a QuikChange kit (Stratagene, United States). The DNA sequences of all constructs were confirmed by the DNA sequence analysis. BCAB and its mutant forms were expressed in *Е. coli* cells and isolated as described elsewhere (Armstrong et al., 1966; Dolgikh et al., 1984).

### Determination of the amount (number) of contacts for amino acid residues

To analyze the structure and calculate the number of contacts for every amino acid residue in protein BCAB, we used RasWin (v.2.7 Herbert J. Bernstein) and DSSP (Kabsch and Sander, 1983). The structure of protein PDB ID: 1V9E was used in these analyses. When calculating the number of contacts, two atoms were accepted to be in contact if the distance between them (r) did not exceed 5 Å. The distribution of the residue-residue contacts doesn’t change essentially if to use contact distance 4 or 6 Å.

### Unfolding and refolding experiments

For refolding studies, samples of unfolded BCAB (solution containing 9.5 M urea, 20 mM phosphate buffer, pH 8.0) were prepared at high protein concentrations (usually 2–10 mg/ml) and incubated in 9.5 M urea for 24 h in order to reach complete unfolding. Refolding experiments were initiated by 10–100-fold dilution of the unfolded protein into the phosphate buffer (20 mM phosphate buffer, pH 8.0) containing desired urea concentrations. The final protein content in the refolding mixtures varied from 0.01 to 1.0 mg/ml. Experiments on the BCAB unfolding were carried out in a similar manner, by 10–100-fold dilution of the native protein to solutions containing desired urea concentrations in 20 mM phosphate buffer, pH 8.0. Fluorescence unfolding and refolding kinetics were monitored using a Cary Varian Eclipse spectrofluorometer (Agilent Technologies, Australia) equipped with a temperature-controlled holder. The samples were excited at 280 nm and the emission was monitored at 355 and 335 nm using the cuvette with 10 mm × 10 mm path lengths. Slits of 2 nm were applied for excitation and emission. All measurements were conducted in 20 mM NaPO4 buffer at pH 8.0, 20°C. All obtained kinetic curves were well described by a double or three-exponential approximation. Kinetic data were repeated five times for each point.

### Apomyoglobin

Based on the plasmid containing a wild-type (WT) apomyoglobin gene, plasmids possessing mutant apomyoglobin gene variants with selected substitutions were derived. Recombinant proteins were isolated and purified according to the methods described in our previous studies (Baryshnikova et al., 2005b; Baryshnikova et al., 2005a; Baryshnikova et al., 2005c; Baryshnikova et al., 2007).

Kinetic measurements of apomyoglobin were taken using a spectrofluorometer Chirascan Spectrometer (Applied Photophysic, United Kingdom) equipped with a stopped-flow attachment. The excitation wavelength was 280 nm, and emission spectra were recorded using a 320-nm cut-off glass optical filter. The initial urea concentration was 5.5 M for refolding experiments, and 0.0 M for unfolding ones. The initial protein solution was mixed (1:1) with a buffer of various urea concentrations using the stopped-flow attachment. The final protein concentration was 0.03 mg/ml. All the measurements were carried out at 11°C in 20 mM sodium phosphate buffer, pH 6.2. Unfolding/refolding kinetic curves at different urea concentrations (0–5.5 M) were obtained for each protein variant. The technique of the measurements was described in detail in our previous works (Baryshnikova et al., 2005b; Samatova et al., 2010).

In this work, we have used urea to unfold proteins, but guanidine hydrochloride or temperature can be used for the same purpose. Using guanidine hydrochloride, it is necessary to introduce a correction along the concentration axis, and in temperature studies, plot graphs in Arenius coordinates.

The measurement technique we used (Baryshnikova et al., 2005b; Samatova et al., 2010) made it possible to exclude the effect of aggregation on the results obtained.

### Selection of apomyoglobin mutations

For substitutions described in this work, hydrophobic amino acids located on the surface of the protein (accessible surface areas were calculated using the DSSP) were chosen. Substitutions of two, three, and four amino acids (Figure 2) were carried out so that all these substitutions were concentrated in one region of the amino acid sequence of the protein and in its 3D structure in order to maximize the effect on the molten globule state, if such an effect were observed.

Amino acids H36 and F106 were chosen to be substituted with cysteines. The distance between С_β_ atoms of these amino acid residues is 4.8А in the PDB:1bzp structure. Amino acids were selected that were maximally distant along the chain, but close together in the folded protein. Amino acids located in the F helix of the protein (this part of the protein is not structured in the apo form) and at the N-terminus of the protein were excluded due to the high mobility of these regions of the protein (Jennings and Wright, 1993).

Сysteines oxidation and cysteine bridge formation were verified for the H36C-F106C mutant form of apomyoglobin. The pure protein was dissolved in 0.2 M Tris-HCl, pH 7.5, 0.2 M NaCl, 1 mM EDTA to a protein concentration of 5 mg/ml. The protein was oxidized by addition of oxidized and reduced glutathione to final concentrations of 10 and 2 mM, respectively. After 24 h incubation at room temperature, glutathione was removed with a PD-10 Desalting column. Then the quantity of free SH groups was defined using the Ellmans reagent (Zahler and Cleland, 1968). Additional checks were performed by electrophoresis. A protein crosslinked with an ss-bond changes its mobility in the gel if reducing agents are not added to SDS electrophoresis. Thus, we additionally controlled the formation of bonds between cysteines by electrophoresis.

#### Analysis of the chevron plots

Rate constants of the transition over the rate-limiting free energy barrier between *I* and *N* (*k*_*NI*_ for the *N→I* transition and *k*_*IN*_ for *I→N*) are considerably less than the *I↔U* rates *k*_*UI*_ and *k*_*IU*_ (here, about 1 s^−1^ and 10^−3^ s^−1^, respectively), thereby the experimentally observed rate constants can be described by the equation (Parker et al., 1995; Baldwin, 1996).

$${\text{k}}_{\text{obs}}={\text{k}}_{\text{NI}}+{\text{f}}_{\text{I}}\cdot {\text{k}}_{\text{IN}},$$ (1)

where f_I_ is an intermediate state population proportional to the amplitude of the burst-phase of refolding kinetics (Figure 2).

#### Plotting of the free-energy profiles

The rate constants of the fast *I↔U* transition (*k*_*UI*_ and *k*_*IU*_) cannot be measured experimentally because this event occurs within the stopped-flow dead time. Nevertheless, relative positions of free energies for *U, I, N*, and *TS* states (*F*_*U*_ *, F*_*I*_ *, F*_*N*_ and *F*_*TS*_, respectively) can be estimated over the entire range of urea concentrations using the experimentally measured rate constants for protein folding/unfolding (*k*_*obs*_) and percentage of population of the *I* state (*f*_*I*_).

*F*_*I*_ *-F*_*U*_ can be obtained, at various urea concentrations *M*, as

$${\text{F}}_{\text{I}}\left(\text{M}\right)-{\text{F}}_{\text{U}}\left(\text{M}\right)=-\text{RT}\cdot \text{ln}\frac{{\text{f}}_{\text{I}}\left(\text{M}\right)}{1-{\text{f}}_{\text{I}}\left(\text{M}\right)}$$ (2)

*F*_*N*_ *-F*_*I*_ can be obtained from the *N↔I* two-state transition (Fersht, 2000b) as

$${\text{F}}_{\text{N}}\left(\text{M}\right)-{\text{F}}_{\text{I}}\left(\text{M}\right)=-\text{RT}\cdot \text{ln}\frac{{\text{k}}_{\text{IN}}\left(\text{M}\right)}{{\text{k}}_{\text{NI}}\left(\text{M}\right)},$$ (3)

where *k*_*NI*_(*M*) is the unfolding rate and *k*_*IN*_(*M*) is the refolding rate constants extrapolated to urea concentration *M*.

The rate constant *k*_*NI*_ is determined by the *F*_*TS*_ *-F*_*N*_ difference (*see* the transition state theory (Fersht, 2000a; 2000b)) so, *F*_*TS*_ *-F*_*N*_ can be obtained as:

$${\text{F}}_{\text{TS}}\left(\text{M}\right)-{\text{F}}_{\text{N}}\left(\text{M}\right)=-\text{RT}\cdot \left[\text{ln}\left[{\text{k}}_{\text{NI}}\left(\text{M}\right)\right]-\text{ln}\left(\frac{\text{RT}}{\text{h}}\right)\right]$$ (4)

where $\text{RT}\cdot \text{ln}\frac{\text{RT}}{\text{h}}=69\text{.}4\text{kJ/mol}$ is the constant at 11°C (284 K).

#### Determination of the population of the kinetic intermediate

The kinetic intermediate (molten globule state) population among the nonnative states, i.e., *f*_*I*_ *(M) = [I]/([I] + [U])*, where *[I]* and *[U]* are concentrations of the *I* and *U* states, was determined at various urea concentrations *M* from the burst phase fluorescence amplitude,

$$f_{I}\left(M\right)=\frac{A\left(M\right)-A_{U}\left(M\right)}{A_{I}\left(M\right)-A_{U}\left(M\right)}$$ (5)

where *A*_*U*_ *(M)* and *A*_*I*_ *(M)* are the integral fluorescence intensities for *U* and *I* states, and *A(M)* is the integral fluorescence intensity achieved as a result of the first, burst *(U → I)* phase in the *(U → I)* transition in protein folding at the given final urea concentration *M*.

## Data Availability

The original contributions presented in the study are included in the article/Supplementary Material, further inquiries can be directed to the corresponding author.
